# *Streptococcus Salivarius*: A Potential Salivary Biomarker for Orofacial Granulomatosis and Crohn’s Disease?

**DOI:** 10.1093/ibd/izz022

**Published:** 2019-02-23

**Authors:** Rishi M Goel, Erica M Prosdocimi, Ariella Amar, Yasmin Omar, Michael P Escudier, Jeremy D Sanderson, William G Wade, Natalie J Prescott

**Affiliations:** 1 Department of Gastroenterology, Kingston Hospital, London, United Kingdom; 2 Centre for Host-Microbiome Interactions, Faculty of Dentistry, Oral & Craniofacial Sciences, King’s College London, London, United Kingdom; 3 Department of Medical and Molecular Genetics, Faculty of Life Science and Medicine, King’s College London, London, United Kingdom; 4 Oral Medicine Unit, Faculty of Dentistry, Oral & Craniofacial Sciences, King’s College London, London, United Kingdom; 5 Department of Gastroenterology, Guy’s & St. Thomas’ Hospitals NHS Foundation Trust, London, United Kingdom

**Keywords:** Orofacial granulomatosis, microbiota, saliva, *Streptococcus*

## Abstract

**Background:**

Orofacial granulomatosis (OFG) is a rare disease characterised by chronic, noncaseating, granulomatous inflammation primarily affecting the oral cavity. Histologically, it is similar to Crohn’s disease (CD), and a proportion of patients have both OFG and CD. The cause of OFG remains elusive, but it has been suggested that microbial interactions may be involved. The aim of this study was to compare the salivary microbial composition of subjects with OFG and/or CD and healthy controls.

**Methods:**

Two hundred sixty-one subjects were recruited, of whom 78 had OFG only, 40 had both OFG and CD, 97 had CD only with no oral symptoms, and 46 were healthy controls. Bacterial community profiles were obtained by sequencing the V1-V3 region of the 16S rRNA gene.

**Results:**

There were no differences in richness or diversity of the salivary bacterial communities between patient groups and controls. The relative abundance of the *Streptococcus salivarius* group was raised in patients with OFG or CD only compared with controls, whereas that of the *Streptococcus mitis* group was lower in CD compared with both OFG and controls. One *S. salivarius* oligotype made the major contribution to the increased proportions seen in patients with OFG and CD.

**Conclusions:**

The salivary microbiome of individuals with OFG and CD was similar to that found in health, although the proportions of *S. salivarius*, a common oral *Streptococcus*, were raised. One specific strain-level oligotype was found to be primarily responsible for the increased levels seen.

## INTRODUCTION

Orofacial granulomatosis (OFG) is a rare chronic disease characterized by lip swelling and oral inflammation. The term was originally used to describe oral signs clinically and histologically resembling Crohn’s disease (CD) in patients with no apparent disease elsewhere in the gastrointestinal tract.^1^ Crohn’s disease is a chronic, granulomatous, inflammatory condition that can affect any part of the gastrointestinal tract but most commonly occurs in the terminal ileum. As the mouth is continuous with the gastrointestinal tract and can be affected by granulomatous inflammation, the term “oral CD” has also been used to describe patients with granulomatous inflammation of the oral cavity.

In addition, although a majority of patients with OFG without gut symptoms have been shown to have microscopic intestinal granulomata, only a relatively small proportion develop gut CD.^2, 3^ Because it is a rare disorder, the reported geographical distribution of OFG may be skewed by differences in disease classification and reporting. Notwithstanding this, the majority of cases have been reported in the United Kingdom—particularly Scotland—and OFG seems to occur in greater frequency with concurrent CD in Northern Europe as compared with the South.^4^ Males and females seem to be affected equally, with the median age of disease onset being 23 years.^2^

The clinical features of OFG include recurrent lip swelling and oral ulceration. Persistent inflammation can result in disfiguring fibrotic disease which, in some cases, causes permanent lip swelling refractory to medical therapy that requires debulking surgery. Other features include gingival erythema,^5^ mucosal tags, and “cobblestoning” caused by buccal oedema.^6^ Being facially disfiguring, the disease carries a significant psychological burden for affected individuals.^7^

The underlying cause of OFG remains unknown, but it is likely to have a multifactorial aetiology. Patients with OFG have a high incidence of atopy compared with the general population,^8, 9^ and dietary antigens including cinnamon and benzoate compounds may trigger disease exacerbations. Excluding these antigens from the diet has been found to be effective in controlling OFG in up to 25% of individuals and is the first line of intervention in management.^10, 11^ Immunohistochemistical analysis of oral biopsies from patients with active OFG identified a novel population of subepithelial dendritic B cells that expressed IgE,^12^ supporting the concept that an antigen may trigger an immediate hypersensitivity reaction.

Other granulomatous diseases such as sarcoidosis or tuberculosis are known to result from infection by specific microorganisms. It has been suggested that *Mycobacterium avium* s.s. *paratuberculosis* (MAP) might play a similar role in CD but has yet to be definitively proven.^13, 14^ Raised levels of antibodies against a mycobacterial stress protein have been found in patients with OFG,^15^ but MAP has not been detected in OFG lesions.^16, 17^ The spirochete *Borrelia burgdorferi* has also been implicated in OFG based on raised antibody levels to the organism and apparent treatment success with penicillin,^18, 19^ but this finding was not confirmed in a later study.^20^

To date, there is no compelling evidence for the role of a specific infective organism in OFG. It is possible, however, that the initiating event for OFG is an inappropriate immune response to a member or members of the normal microbiota giving rise to inflammation. This would change the local environment by altering oral surfaces and thereby changing colonization patterns and/or providing serum-derived nutrients that enhance the growth of secondary colonizers. The aim of the study was to use 16S rRNA gene community profiling to determine the composition of the salivary microbiome in patients with OFG only, OFG with concurrent CD, and compare this with patients with CD without oral involvement and with healthy controls.

## MATERIALS AND METHODS

### Patients and Controls

Patients attending a specialist OFG clinic in the department of Oral Medicine at Guy’s & St. Thomas’ Hospitals, London, were recruited over a 2-year period. Patients with CD were recruited via IBD clinics at Guy’s & St. Thomas’ Hospitals as previously described.^21^ Control subjects were recruited from healthy (no disease) volunteers at Guy’s & St. Thomas’ Hospitals. Control subjects were excluded from participating if they reported a history of chronic inflammatory disorder including IBD, current gastro-intestinal symptoms, or oral disease. Two hundred sixty-one subjects were recruited for the study: 40 (18 female) had both OFG and CD (OFG+CD), 78 (43 female) had oral manifestations only (OFG only), 97 (62 female) were diagnosed with Crohn’s disease without any oral symptoms (CD only), and 46 (33 female) were healthy controls (HC). The age of the subjects at the time of collection ranged from 16 to 79 years. Each patient provided informed verbal and written consent.

The primary inclusion criterion for patients was a confirmed history of active or inactive OFG and/or CD. The diagnosis of OFG was based on clinical features including lip swelling and typical oral ulceration. Where available, histology results were also used to support the diagnosis. The diagnosis of CD was based on conventional clinical, biochemical, endoscopic, histological, and radiological criteria. Patients were excluded from the study if they were being treated with antibiotics at the time of sampling.

All patients underwent an oral examination, and the sites of involvement and severity of OFG were recorded as part of a standardized oral disease activity score (ODAS). Other oral findings were also recorded, particularly dental disease, active carious disease, and other oral mucosal changes. Where possible, patients, with their consent, underwent a basic periodontal examination (BPE) to assess for gingival disease. In the BPE, the mouth was divided into sextants, and each sextant scored from 0 to 4 with 0 indicating no pocketing or bleeding in that sextant and 4 indicating advanced periodontitis. The scores for each sextant were summed to give a value between 0 and 24. Basic periodontal examination scores were compared between groups. This variable was transformed by categorical grouping into 3 classes, and the differences in microbiome composition between BPE classes were assessed. Subject groups were compared for age and gender distribution. The effect of immunosuppressant therapy and antitumor necrosis factor (TNF)-α therapy on the microbiome of subjects with OFG was determined.

The study was approved by the local Research Ethics Committee (Approval No. 12/YH/0172; Yorkshire & The Humber REC).

### Sample Collection

Whole saliva was collected by asking patients or volunteers to spit in a universal container until a minimum volume of at least 1 mL had been obtained. Saliva samples were immediately placed on ice and then transferred within 3 hours to a freezer for storage at −70°C. All samples were anonymized and coded.

### Bacterial Community Profiling

DNA was extracted from the saliva samples by means of the Genelute DNA extraction kit (Sigma-Aldrich), and 16S rRNA genes were amplified by polymerase chain reaction (PCR) with primers 27F (with the YM modification) and 519R.^22, 23^ The primers incorporated a unique barcode and Roche 454 adapters. Polymerase chain reaction amplicons were purified, sized, quantified, and pooled in equimolar proportions. Emulsion PCR and unidirectional sequencing of the libraries were performed using the Lib-L kit and Roche 454 GS-FLX Titanium sequencer.

### Data Analysis

Preprocessing and analysis of sequences were carried out using the mothur analysis suite version 1.36.1^24^ based on the Schloss SOP (January 2016). Initial denoising was performed using AmpliconNoise algorithm. Subsequently, any sequences less than 440 bases in length or that had >2 mismatches in the primer, >1 mismatch in barcode regions, and homopolymers of >8 bases were removed from the dataset. The remaining sequences were trimmed to remove the primers and barcodes and aligned to the SILVA 16S rRNA reference alignment.^25^ An additional precluster step was performed in mothur to merge sequences with 4 or fewer bases differences. The UChime algorithm,^26^ as implemented by mothur, was used to identify sequence chimeras, which were removed from the analysis. Sequences were clustered into Operational Taxonomic Units (OTUs) at a sequence dissimilarity distance of 0.015 using an average neighbor algorithm and then classified using a Naïve Bayesian classifier implemented in mothur with the Human Oral Microbiome Database reference dataset v13.2.^27^ The α-diversity of bacterial communities based on OTUs was analyzed using approaches implemented by mothur: richness of the communities was assessed by the number of observed OTUs and the Chao1 richness index; diversity of the communities was estimated using the Simpson inverse diversity index. Richness and diversity estimates were compared between groups using Kruskal Wallis test.

To compare the β diversity of samples based on OTUs, the thetaYC metric, which compares community structure by accounting for the relative abundance of taxa,^28^ was used to generate distance matrices in mothur. Analysis of Molecular Variance (AMOVA),^29^ as implemented by mothur, was then performed to determine if any differences between the microbiomes of the experimental groups were statistically supported by differences in the distance matrix. LeFSE^30^ was used to identify OTUs differentially abundant between groups.

### Minimum Entropy Decomposition (Oligotyping)

Minimum entropy decomposition was performed on the same samples used for the alpha and beta diversity analyses, excluding the 19 samples falling below 3076 sequences. Denoising, alignment, chimera removal, and taxonomy assignation were performed using the mothur analysis suite,^24^ as described previously. Sequences identified as belonging to the genus *Streptococcus* were then extracted and formatted using the “mothur2oligo” tool (available at https://github.com/michberr/MicrobeMiseq/tree/master/mothur2oligo).

Minimum entropy decomposition analysis^31^ was performed using MED pipeline version 2.1 (available at http://oligotyping.org/). The MED algorithm is similar to the previously described oligotyping algorithm^32^ and differentiates taxa on the basis of single-nucleotide differences in the positions of highest entropy. The parameters used were minimum substantive abundance of a MED node (-M) = 7 and maximum variation allowed in each node (-V) = 4 nt. The total number of *Streptococcus* sequences analyzed was 564,342. Of these, 62,706 were removed as outliers due to the minimum substantive abundance parameter (-M, set to 60), and 8262 were removed as outliers due to the maximum variation at each node parameter (-V, set to 5). Thus, after the refinement, 493,374 were analyzed and classified into 370 MED nodes (oligotypes). Sequences representative of each oligotype were identified at species level by comparison with the Human Oral Microbiome database through the BLAST web tool, accessible at http://www.homd.org.

## RESULTS

**FIGURE 1.**
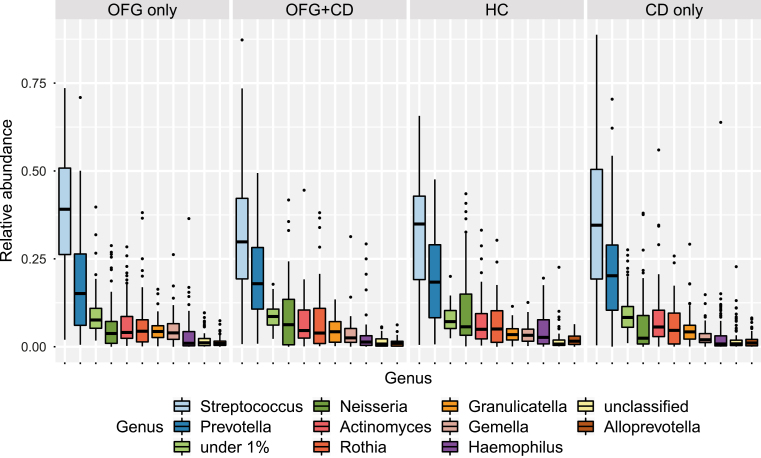
Box plot showing relative abundance of predominant bacterial genera by patient group. The group “under_1%” combines all genera present at less than 1% relative abundance.

A total of 1,630,578 sequences were obtained after denoising and quality filtering. Figure 1 shows the predominant bacterial genera found in the samples by group. For most individuals, the communities were dominated by the genera *Streptococcus* and *Prevotella*, although some subjects had no or very few streptococci.

The data were normalized by subsampling to a level of 3076 sequences per sample, which excluded 19 samples from the subsequent analyses. Subject group sizes for the subsequent analyses were as follows: OFG only (74), OFG+CD (38), CD only (85), and HC (45). There were no significant differences between the groups for the Chao1 or inverse Simpson indices, nor for the average number of observed OTUs (Table 1, Kruskal Wallis test).

**TABLE 1.** Richness and Diversity of the Salivary Microbiota in Subject Groups

Subject Group	n	Observed OTUs	Chao1	Inverse Simpson
		(sd)	(sd)	(sd)
OFG only	74	198.6	505.1	11.2
		54.2	152.5	6.2
OFG+CD	38	183.4	449.5	9.7
		57.2	125.6	4.8
HC	45	196.1	496.3	11.0
		53.5	161.1	4.6
CD only	85	203.5	493.4	9.2
		57.5	133.6	5.1

The composition of the microbial communities among the 4 subject groups was significantly different (AMOVA, *P* value < 0.001). Pairwise comparisons of the individual subject groups were performed, and CD seemed to be the primary driver of intergroup differences. For example, the CD only group was significantly different from both the HC group (*P* < 0.001; significance threshold using Bonferroni correction: 0.008) and the OFG only group (*P* < 0.001). The OFG+CD group was significantly different from the HC group (*P* = 0.006), whereas OFG only was not significantly different from the HC group.

Mean age and gender distribution were not found to be significantly different between the 4 groups (age: Kruskal Wallis test; gender distribution: χ^2^ test). Neither immunosuppressive nor anti-TNF-α therapy had a significant effect on microbiome composition in subjects with OFG (OFG only and OFG+CD groups combined) (AMOVA).

**FIGURE 2.**
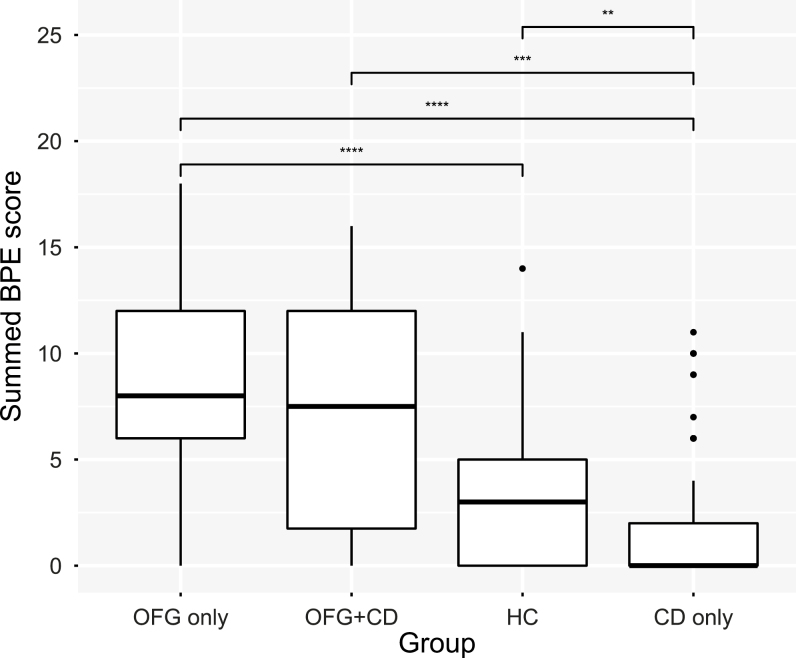
Box plot showing summed BPE scores as a proportion of the total microbiota. Upper and lower edges of the boxes are the first and third quartiles; the line inside the box is the second quartile (median); individual dots are outliers (** *P* < 0.01; *** *P* < 0.001; **** *P* < 0.0001, Kruskal Wallis test)

As oral disease status is known to be a major factor influencing oral microbiome composition, the basic periodontal examination was used to assess the subjects’ gingival health. Basic periodontal examination scores were recorded for 207 of the 261 subjects and were significantly different between phenotype groups (*P* < 0.001, Kruskal Wallis), as shown in Figure 2. Overall, there was a clear trend for subjects with OFG only to have higher BPE scores than HC, whereas CD only patients had lower BPE scores. In view of this, the effect of BPE score on microbiome composition was investigated. Basic periodontal examination scores were assigned to 3 class variables: low, ≤2; middle, 2–10; high, ≥10. There was no significant difference in microbial composition between BPE class groups by AMOVA.

**FIGURE 3.**
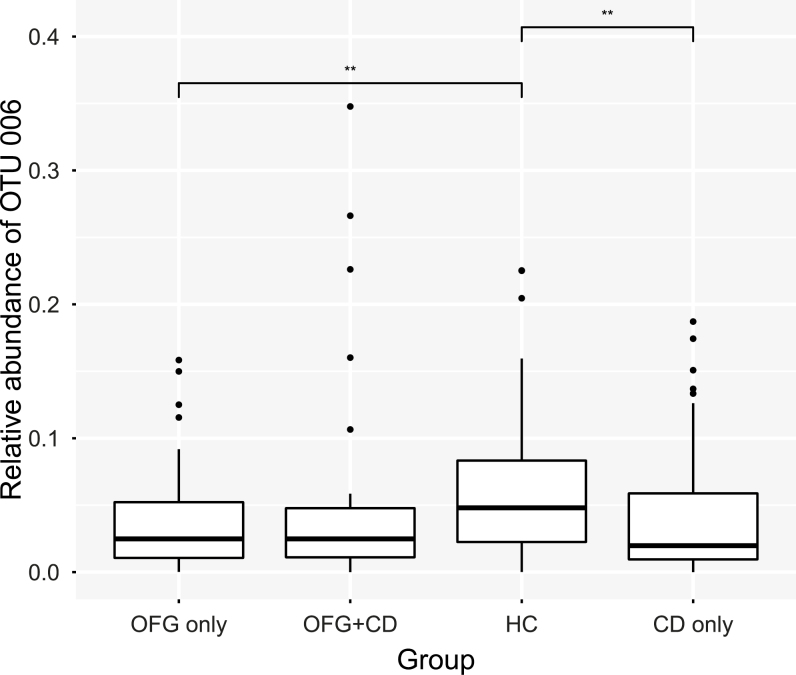
Box plot showing the relative abundance of OTU 6 (*Haemophilus parainfluenzae*) as a proportion of the total microbiota. Upper and lower edges of the boxes are the first and third quartiles; the line inside the box is the second quartile (median); individual dots are outliers (** *P* < 0.01, Wilcoxon test).

The OTUs responsible for the differences in microbial composition seen between groups are shown in Table 2. The default threshold of 2 was used for the logarithmic LDA score for discriminative features. Eleven OTUs were found to be differentially represented between groups. Seven of these overrepresented OTUs were over-represented in the HC group, suggesting that the disease phenotypes were associated with loss of normal microbiota components. Most of the differentially represented OTUs were of relatively low abundance, with only 3 of them (OTU 1, 2, and 6) present in the dataset at a relative abundance of greater than 0.01. Operational taxonomic unit 6 was identified as *Haemophilus parainfluenzae*, and its relative abundance was significantly reduced in OFG only and CD only compared with HC (Fig. 3). Operational taxonomic units 1 and 2 were identified as unclassified members of the genus *Streptococcus* and were the most frequently detected OTUs in the study, making up 17.3% and 7.8%, respectively, of the oral bacterial community across all subjects. Because *Streptococcus* species vary widely in the roles that they play in oral ecology and disease, minimum entropy decomposition (MED) was used to lend more precision to species-level identification.

**TABLE 2.** OTUs Over-represented in Subject Groups (LeFSE)

Group	OTU	Species Over-represented	Mean Relative Abundance_a_	Log LDA
OFG only	152	*Catonella morbi*	0.0003	2.6
OFG+CD	065	*Lachnoanaerobaculum* unclassified	0.001	2.9
HC	002	*Streptococcus* unclassified (*mitis* group)	0.78	4.5
	006	*Haemophilus parainfluenzae*	0.044	4.1
	032	*Prevotella sp.* HOT-299	0.003	3.3
	037	*Alloprevotella tannerae*	0.002	3.1
	067	*Bergeyella sp.* HOT-322	0.0009	2.8
	141	*Neisseria elongata*	0.0004	2.6
	162	*Prevotella sp.* HOT-305	0.003	2.7
CD only	001	*Streptococcus* unclassified (*salivarius* group)	0.173	4.9
	047	*Fusobacterium nucleatum* subsp. *vincentii*	0.001	2.9

^a^all samples

**FIGURE 4.**
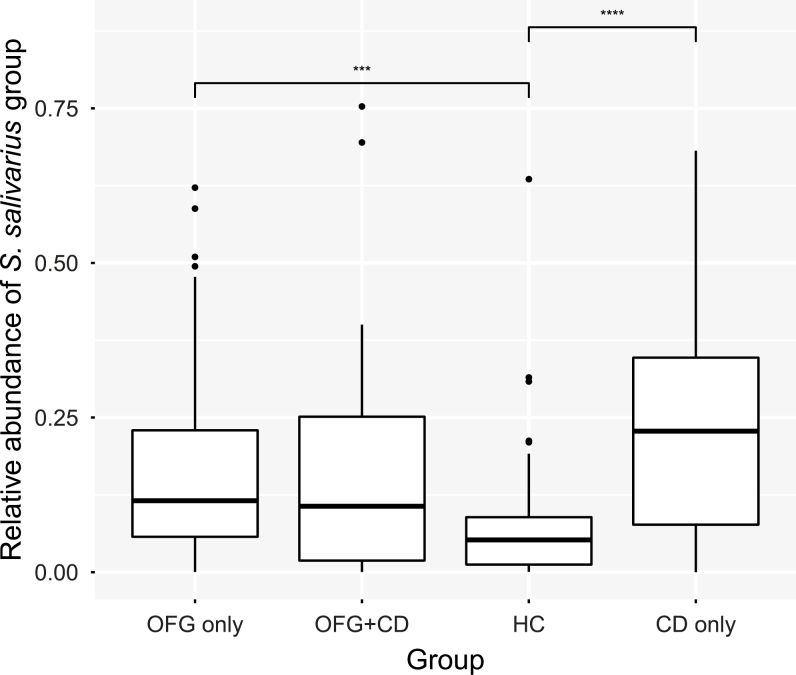
Box plot showing summed *S. salivarius* group oligotypes as a proportion of the total microbiota. Upper and lower edges of the boxes are the first and third quartiles; the line inside the box is the second quartile (median); individual dots are outliers (*** *P* < 0.001; **** *P* < 0.0001, Wilcoxon test).

**FIGURE 5.**
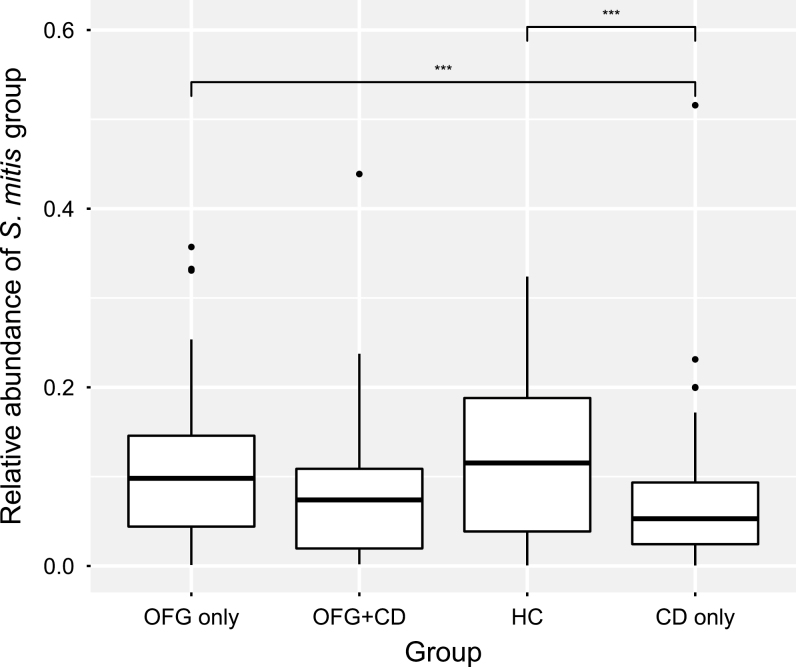
Box plot showing summed *S. mitis* group oligotypes as a proportion of the total microbiota. Upper and lower edges of the boxes are the first and third quartiles; the line inside the box is the second quartile (median); individual dots are outliers (*** *P* < 0.001, Wilcoxon test).

**FIGURE 6.**
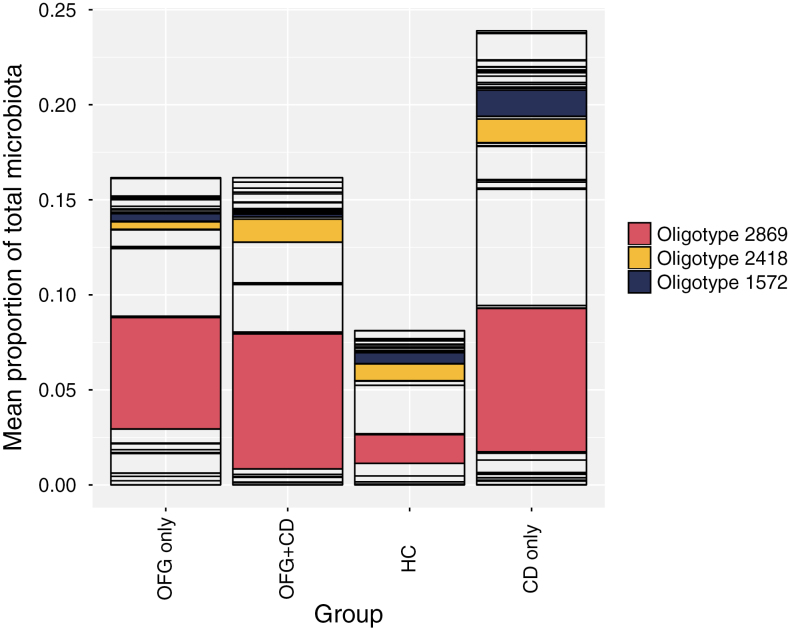
Proportions of individual *S. salivarius* group oligotypes in subject groups. Oligotypes highlighted in color showed significant differences between groups (Kruskal Wallis test).

The *Streptococcus* sequences were binned into 370 oligotypes that were subsequently grouped by species after BLAST interrogation of the HOMD database. Where multiple species-level BLAST identifications were above 98.5% sequence identity, the identification was made to a group of species. The mean abundances of species and species groups making up more than 1% of the total microbiota were compared between groups, using Wilcoxon test with Bonferroni correction for multiple testing. *Streptococcus salivarius* proportions were significantly higher in the OFG only and CD only groups compared with HC (Fig. 4). The *S. mitis* group also showed significant differences among groups (Fig. 5), with healthy HC and OFG only having significantly higher relative abundances than CD only. In addition, the relative abundances of individual oligotypes whose mean relative abundance was over 0.5% were compared across the groups. Three *S. salivarius* oligotypes were found to show significant differences between groups (Fig. 6). Oligotype 2869 showed the largest differences with the OFG only, CD only, and OFG+CD groups all having significantly higher relative abundance than the HC group.

## DISCUSSION

The results of this study indicate that the oral microbiome in subjects with orofacial granulomatosis is not markedly different from that of healthy controls or subjects with Crohn’s disease because there were no differences in richness or diversity between the groups and all subjects had a typical oral microbiome compositional profile. This finding is in contrast to the results of numerous studies looking at the effect of CD on the composition of the intestinal microbiome.^33^ The fecal and mucosal microbiome is substantially altered in patients with CD. Richness is reduced,^34, 35^ and the phylum *Firmicutes* is relatively depleted, particularly anaerobes from the order *Clostridiales*, but there are raised proportions of *Proteobacteria*, mainly *Enterobacteriaceae*. The shift toward a less anaerobic bacterial community is thought to be the result of increased levels of reactive oxygen species produced as a part of the inflammatory response.^34^ Specific genus-level microbial signatures of CD have been reported to be reduced levels of *Faecalibacterium*, an unknown *Peptostreptococcaceae*, *Anaerostipes*, *Methanobrevibacter*, an unknown *Christensenellaceae* and *Collinsella*, and increased proportions of *Fusobacterium* and *Escherichia*.^36^

Patients with OFG were found to have higher BPE scores than controls, although there was no corresponding difference in microbiome composition. The levels of BPE scores seen were indicative of some degree of gingival inflammation and may have been due to poorer oral hygiene in the patients due to the discomfort caused by the OFG lesions.

The lack of substantial alteration of the oral microbiome in OFG with or without gut CD most likely reflects the fact that the oral microbiome is extremely stable and not greatly affected by diet^37^ or administration of antibiotics.^38^ There were, however, some OTUs which showed differences in relative abundance between groups in the LEfSe analysis. The high number of comparisons performed in microbiome studies when OTU relative abundances are compared between patient groups can lead to spurious associations being revealed by chance, even when significance thresholds, as here, are corrected for multiple comparisons. Such associations should therefore be interpreted with caution. Of the 11 OTUs over-represented in particular subject groups, 7 were in the control group. This suggests that the major shift in OFG and CD was the relative loss of normal microbiota taxa. Another important consideration in interpreting OTU association analyses is whether the size of the effect is biologically significant. Many of the OTUs found to be differentially abundant were present at extremely low levels, and therefore, only those present at a relative abundance of greater than 1 % were considered further. Levels of *H. parainfluenzae* were reduced in all patient groups compared with controls. *Haemophilus parainfluenzae* is a commonly occurring member of the normal microbiota, and the significance of this finding is unclear. In contrast, proportions of oligotypes belonging to the *S. salivarius* group were found to be significantly raised in the OFG only and CD only groups. *Streptococcus salivarius* and related species are regarded as health-associated and are found primarily on the dorsum of the tongue and the pharyngeal mucosa.^39, 40^ Indeed, strains of *S. salivarius* are used as probiotics with beneficial properties against oral conditions such as halitosis and pharyngitis^41^ and have been shown to have anti-inflammatory properties in vitro via downregulation of the NF-κB pathway.^42^ It is not clear why the proportions of these species should be raised in OFG and CD, but the diseases may change the oral mucosa in ways which promote the adherence and retention of these species. It is particularly interesting that 1 oligotype, 2869, was specifically elevated in subjects with OFG or CD. It is known that strains of a species can differ markedly in their biological and pathogenic properties, and it seems that members of this oligotype, found in multiple subjects, has a particular and numerically strong relationship with OFG. These findings are of particular interest as bacterial antigens, including streptococci, are a known common target for Immunoglobulin E (IgE). And previous studies have identified infiltrates of dendritic B cells in the oral epithelium OFG patients which express surface IgE.^12^ Future work should be focused on confirming the association of specific *S. salivarius* strains with OFG by metagenomic analyses that enable strain differentiation,^43^ together with the isolation of representatives of this oligotype and investigation of its properties of relevance to OFG.

In contrast to *S. salivarius*, *S. mitis* group organisms were present at lower relative abundance in the subjects with CD compared with OFG and controls. *Streptococcus mitis* is the commonest streptococcal species found in the human mouth.^40^ The numbers of this group may have been reduced because proportions of *S. salivarius* were raised, which would have affected their relative abundance.

The results of this study demonstrate that the overall composition of the salivary microbiota in OFG and CD was similar to that of healthy controls but that there were some significant—and interesting—differences in levels of 2 of the commonest groups of oral streptococcal commensals, which warrant further investigation. In particular, *S. salivarius* was increased in both CD and OFG, whereas *S. mitis* decreased in CD only.

## CONCLUSION

It has been previously shown that pathogenic variants in genes known to confer a high-risk for CD are enriched in OFG patients with concurrent intestinal disease (OFG+CD).^44^ Furthermore, we have recently demonstrated that a useful toolkit for predicting intestinal inflammation in individuals at greatest risk for CD can be implemented from a small set of genetic markers, such as these, combined with family and lifestyle risk factors.^45^ The study described here provides the first step in the investigation of the utility of salivary microbial biomarkers as proxy for gastrointestinal dysbiosis and disease. Further studies that can correlate these findings with host genetics, immune status, and metabolomics, and other risk factors could help to develop prediction tools to identify those OFG patients that are at greatest risk of developing intestinal CD, thereby opening up the possibility for early intervention. In addition, a better understanding of the interactions between microbial shifts, inflammation, and disease pathology would have the potential to lead to further targets for drug development and disease management strategies for this complex phenotype.

## Notes

Supported by: This work was supported by the National Institute for Health Research (NIHR) Biomedical Research Centre at Guy’s and St. Thomas’ NHS Foundation Trust and King’s College London and The Wellcome Trust (094491/Z/10/Z).
